# Wearable porous PDMS layer of high moisture permeability for skin trouble reduction

**DOI:** 10.1038/s41598-020-78580-z

**Published:** 2021-01-13

**Authors:** Sunghyun Yoon, Minho Seok, Mookyum Kim, Young-Ho Cho

**Affiliations:** Department of Bio and Brain Engineering, Korea Advanced Institute of Science and Technology (KAIST), 291 Daehak-ro, Yuseong-gu, Daejeon, 34141 Republic of Korea

**Keywords:** Engineering, Materials science

## Abstract

The present research proposes the present porous polydimethylsiloxane (PDMS) layer for the skin trouble reduced daily life skin attachable devices. The present research proposes the new pores forming method in the PDMS by crystallization and dissolution of the citric acid in the PDMS for fabricating high uniform and small size pores. The present porous PDMS layer (i) decreases the pore size 93.2%p and increases the pore size uniformity 425%p compared to the conventional porous PDMS layer of mixing sugars and PDMS; (ii) is able to be fabricated in the thickness of 21–101 µm by spin-coating; (iii) has the 2.2 times higher water vapor transmission rate (947 ± 10.8 g/day•m^2^) compared to the human skin water vapor transmission rate. The present porous PDMS layer reduces the skin trouble effectively by having the high water vapor permeability, therefore is applicable to the human daily-life skin attachable devices.

## Introduction

Currently, the skin attachable human physiological signal monitoring devices lead skin-troubles represented as skin redness and itchiness^1^ by blocking water vapor evaporation on human skin. Therefore, the skin attachable materials with high water vapor transmission rate is required to reduce the skin troubles for the daily time human physiological signal monitoring.

The skin attachable devices commonly use the materials of polydimethylsiloxane (PDMS), polyurethane, polyimide, and parylene-C^2^. The PDMS is representative for the skin trouble reducing material due to the high water vapor transmission rate. However, the water vapor transmission rate of the PDMS (296 g/day•m^2^) in the thickness of 27.7 µm is lower than the water vapor evaporation rate of the human skin (432 g/day•m^2^^3^), thereby becoming a reason of the skin trouble. The water vapor transmission rate of the PDMS is able to be increased by forming porous structures. The present research focuses on the porous PDMS layer to improve the water vapor transmission rate by combining the material property effect and the structural effect.

The conventional researches fabricate the porous PDMS layer by mixing PDMS and: (i) beads (E.g. polystyrene beads) followed by the polystyrene beads dissolution using toluene^4^; (ii) liquid (E.g. water and ethanol) followed by the liquid evaporation^5^; (iii) aqueous solute (E.g. sugar) followed by the solute dissolving using water^6^.

The other methods for porous PDMS structures are based on: (i) the gas forming in the un-cured PDMS^7,8^; (ii) the PDMS curing in the porous metal mold^9^; (iii) the PDMS mixed with beads (e.g., polystyrene beads^4^, poly(ethylene glycol)^10^, micelle^11^) followed by the bead dissolution; (iv) the PDMS mixed with liquid (e.g., water and ethanol^5^) followed by the liquid evaporation; (v) the PDMS mixed with aqueous solute (e.g., sugar^6^, salt^12^) followed by the solute dissolving using water.

The porous PDMS structures formed by gas in the un-cured PDMS are easily obtained by heating. However, the porous PDMS formed by gas are difficult to obtain the structure in the form of layers due to the difficulty of the layer thickness control in spin-coating process. Also, the porous PDMS structures formed by gas have arbitrary porous shapes due to the difficulty of the uniform heating of the whole volume of the PDMS. The porous PDMS cured in the porous metal molds enable to form layer structures. However, the porous PDMS layers require the molds with different thicknesses to obtain the different thicknesses of the porous PDMS layer. The porous PDMS layers formed by beads mixed in PDMS not only achieve the high uniformity of pore sizes but also enable the thin porous PDMS layers by spin coating. However, the porous PDMS layers formed by beads require the high cost materials such as polystyrene, PEG, or micelle beads, the complex fabrication processes of the bead fabrication, and the self-assembly of beads. The porous PDMS layers mixed with liquid have advantages of the high pore size uniformity, the thin porous PDMS layers by spin coating, and the low cost materials of water and ethanol. However, the porous PDMS layers with liquid limit the porosity under 40%. The porous PDMS structures formed by aqueous solutes achieve the porosity over 50% using the low cost pore forming material of sugar or salt. However, the porous PDMS structures formed by aqueous solutes are difficult for applications to the human skin attachable devices because the large centrifugal forces of the spin-coating process result in the large and non-uniform size (70–400 µm) of sugar.

In this research, we propose a novel porous PDMS layer fabrication including the special process of the citric acid crystallization and dissolution to solve the problems of the conventional porous PDMS layer fabrication methods, as follows. The present porous PDMS layer use citric acids as pore forming material, therefore requiring the 0.16% of the material cost of the conventional methods mixing PDMS and beads for the fabrication of the same porosity porous PDMS layer. The present porous PDMS layer fabrication method is able to acquire higher porosity (< 60%) than the conventional method mixing PDMS and liquid, by controlling the mixing ratio of the PDMS and the citric acids. The present porous PDMS layer form pores inside the PDMS by using ethanol and citric acid, instead of water and sugar of the conventional method mixing PDMS and aqueous solute. The ethanol has higher PDMS permeability than water^13^ and the citric acid have higher ethanol solubility^14,15^ than sugar. The present porous PDMS layer form pores by citric acid crystallization in PDMS followed by the citric acid crystal removal using ethanol, thereby having 93.2%p decreased and 425%p increased pore size and pore size uniformity, respectively, compared to those of the conventional method using sugar. The present porous PDMS layer is applicable to the skin attachable device by distributing the uniform and the small size pores in the spin-coated porous PDMS layer thin membrane in the thickness range of 21–101 μm.

Human skin compatibility of the present porous PDMS layer is evaluated based on the water vapor transmission rate. The water vapor occupies the dominant amount of the human skin secretion, thereby the water vapor transmission rate is adaptable to evaluate human skin compatibility. Surface roughness of the present porous PDMS layer is characterized for the attachment reliability of the human physiological sensors on the present porous PDMS layer.

The present research proposes the novel porous PDMS structure fabrication process to enhance the water vapor transmission rate of the skin attachable devices by citric acid crystallization process. The present porous PDMS layer fabrication process enables the spin-coated porous PDMS layer for the skin attachable device applications by not only reducing the pore sizes but also improving the uniformity of the pore sizes. The present porous PDMS layer is experimentally verified having the skin trouble reduction effect by having the water vapor transmission rate over the water vapor evaporation rate of the human skin, thereby applicable to the daily life physiological signal monitoring human skin attachable device.

## Experimental results and discussion

### Fabrication process

The present porous PDMS fabrication use four materials of PDMS, citric acid, ethanol, and toluene. PDMS is the structure material. Toluene is used to dilute PDMS for easy mixing of PDMS and ethanol-citric acid solution. Citric acid is pore forming material of the present porous PDMS layer fabrication. Ethanol plays two roles of the citric acid solvent and the crystallized citric acid removal.

**Figure 1:**
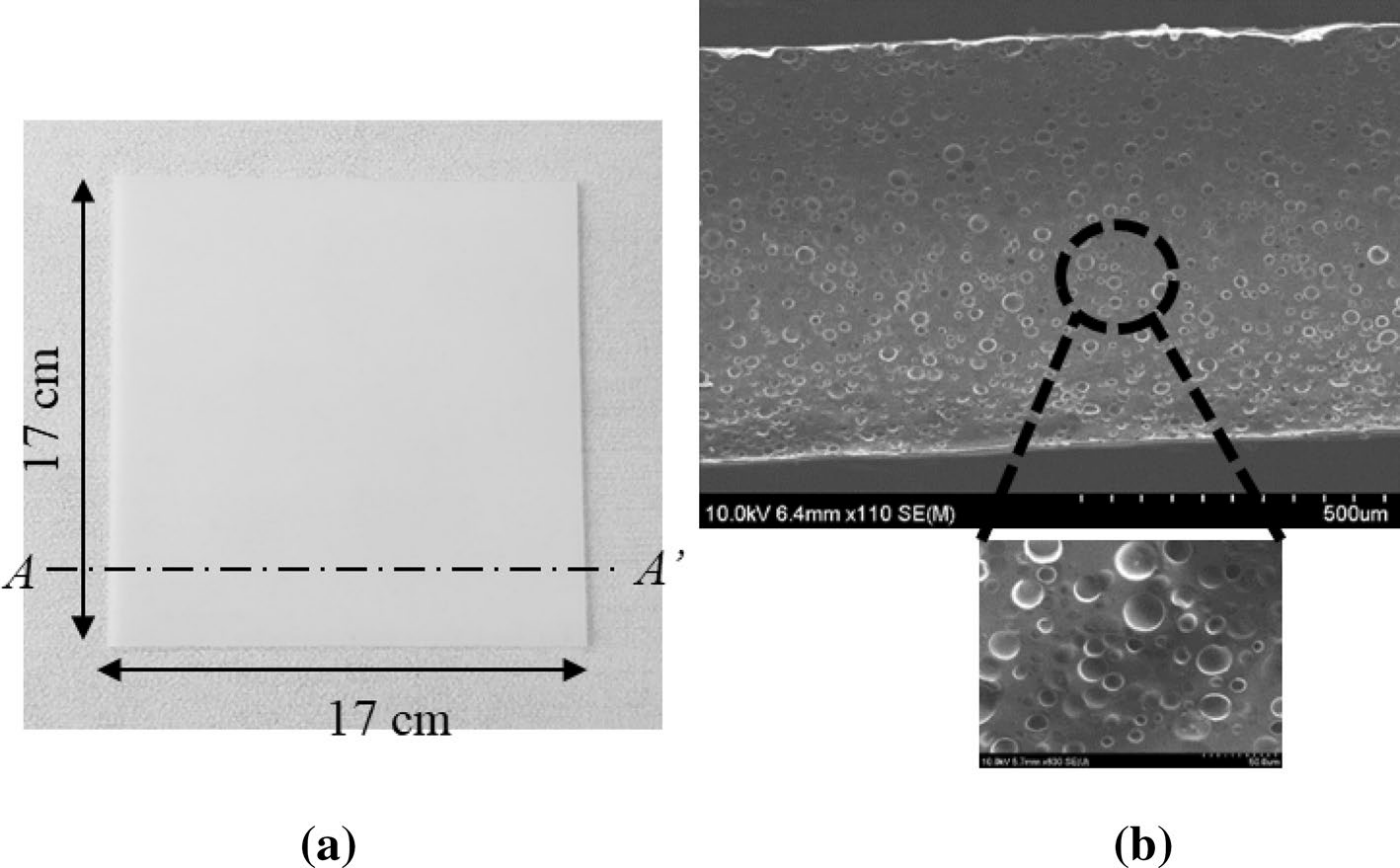
The present porous PDMS layer, fabricated at t_e_ = 150 °C and WMR = 0.5: (**a**) top view; (**b**) SEM of the cross-section A-A’ of figure (**a**).

Figure s1 shows the fabrication process of the present porous PDMS layer. The first step of the present porous PDMS layer fabrication is mixing the four materials of the PDMS, the toluene, the citric acid, and the ethanol (Fig. s1a). The toluene and the ethanol solvent mixture is evaporated on the hot plate to crystallize the citric acid in the PDMS (Fig. s1b). The citric acid crystals and PDMS mixture (Fig. s1c) is molded and cured using the PDMS curing agency (Fig. s1d). The PDMS curing agency is added in the weight ratio of 1:10 with PDMS. The cured citric acid crystals and PDMS mixture is dipped in the ethanol to remove the citric acid crystals (Fig. s1e). The present porous PDMS (Fig. s1f.) is fabricated followed by drying the ethanol. The present porous PDMS layer is fabricated to have different pore size, pore size uniformity, and porosity depending on the two major fabrication conditions of: (i) the toluene and ethanol solvent mixture evaporation temperature (t_e_); (ii) the citric acid and the PDMS weight mixing ratio (WMR). t_e_ controls the evaporation speed of the toluene and the ethanol. The toluene and the ethanol evaporation speed decide the sizes of the citric acid crystals. The sizes of the citric acid crystals affect the pore size and the pore size uniformity of the present porous PDMS layer. The size of the citric acid crystals decides the centrifugal force on each citric acid crystal during the spin-coating membrane fabrication process. Larger citric acid crystals are easy to be removed during the spin-coating process due to the larger centrifugal force. Thus, t_e_ affects the pore size and the pore size uniformity of the present porous PDMS layer, thereby deciding the spin-coating possibility of the porous PDMS layer with the uniformly distributed small-sized pores. t_e_ is controlled in 60, 70, 120, 130, 140, and 150 °C for the present porous PDMS layer fabrication. t_e_ in the range of 80–110 °C are not adaptable to fabricate the present porous PDMS layer. The details about the t_e_ ranges are explained in the method section. WMR decides the porosity of the present porous PDMS layer. The porosity of the porous PDMS layer is decided by the amount of the citric acid crystals removed by the ethanol. The porosity is a dominant factor to decide the water vapor transmission rate of the present porous PDMS layer. Thus, WMR is the key factor to decide the water vapor transmission rate of the present porous PDMS layer. WMR is calculated by the division of the citric acid mass and the PDMS mass. WMR is controlled in the 4 different types of 0.5, 1.0, 1.5, and 2.0 for the present porous PDMS layer fabrication. The specific amount of the materials used in the fabrication process is explained in the method section. Figure 1 shows the present porous PDMS layer, fabricated at t_e_ = 150 °C and WMR = 0.5.

### Volumetric contraction

**Figure 2:**
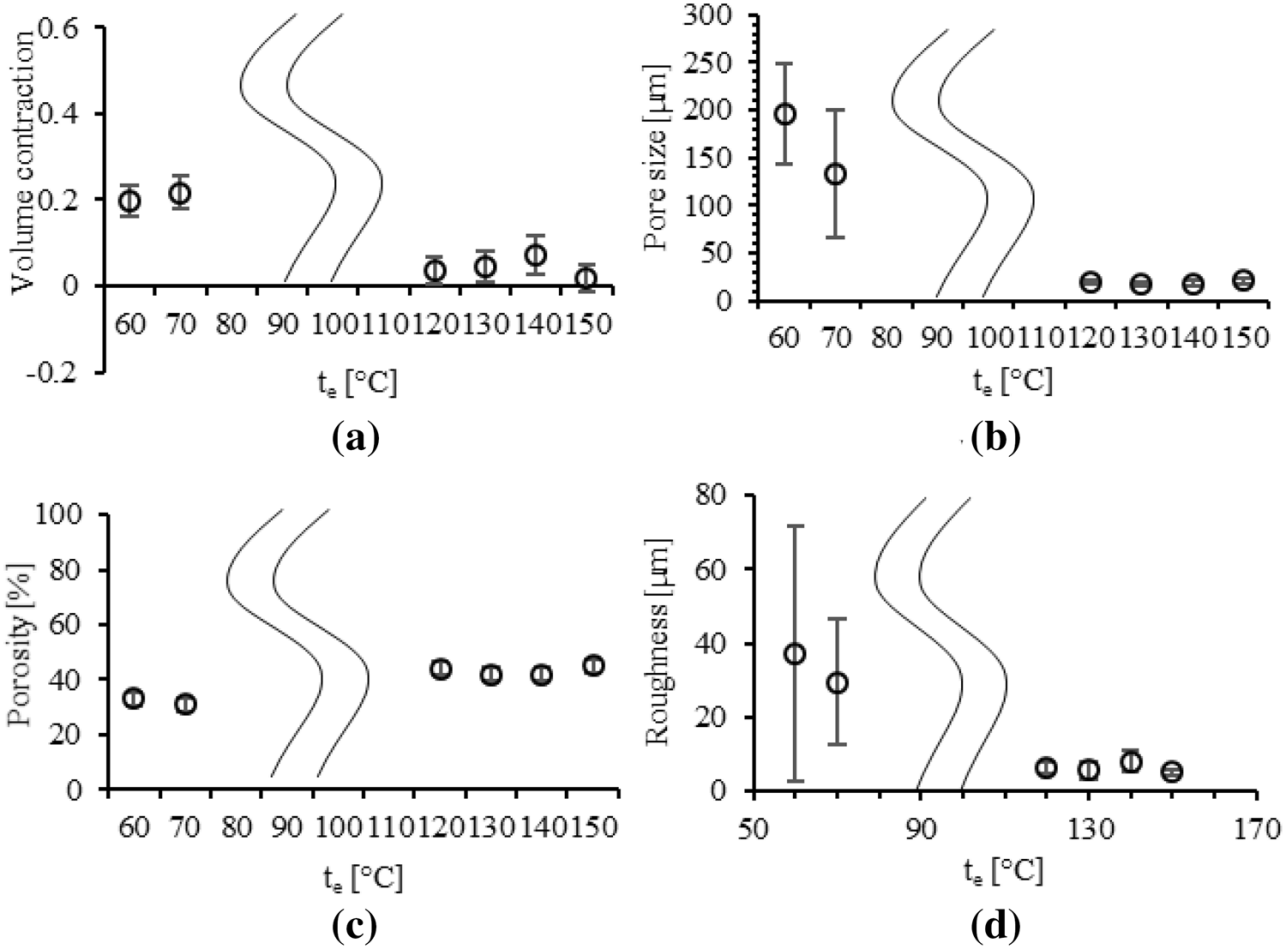
Properties of the present porous PDMS layer, fabricated at WMR = 0.5 for varying t_e_: (**a**) volumetric contraction; (**b**) pore size; (**c**) porosity; (**d**) surface roughness.

**Figure 3:**
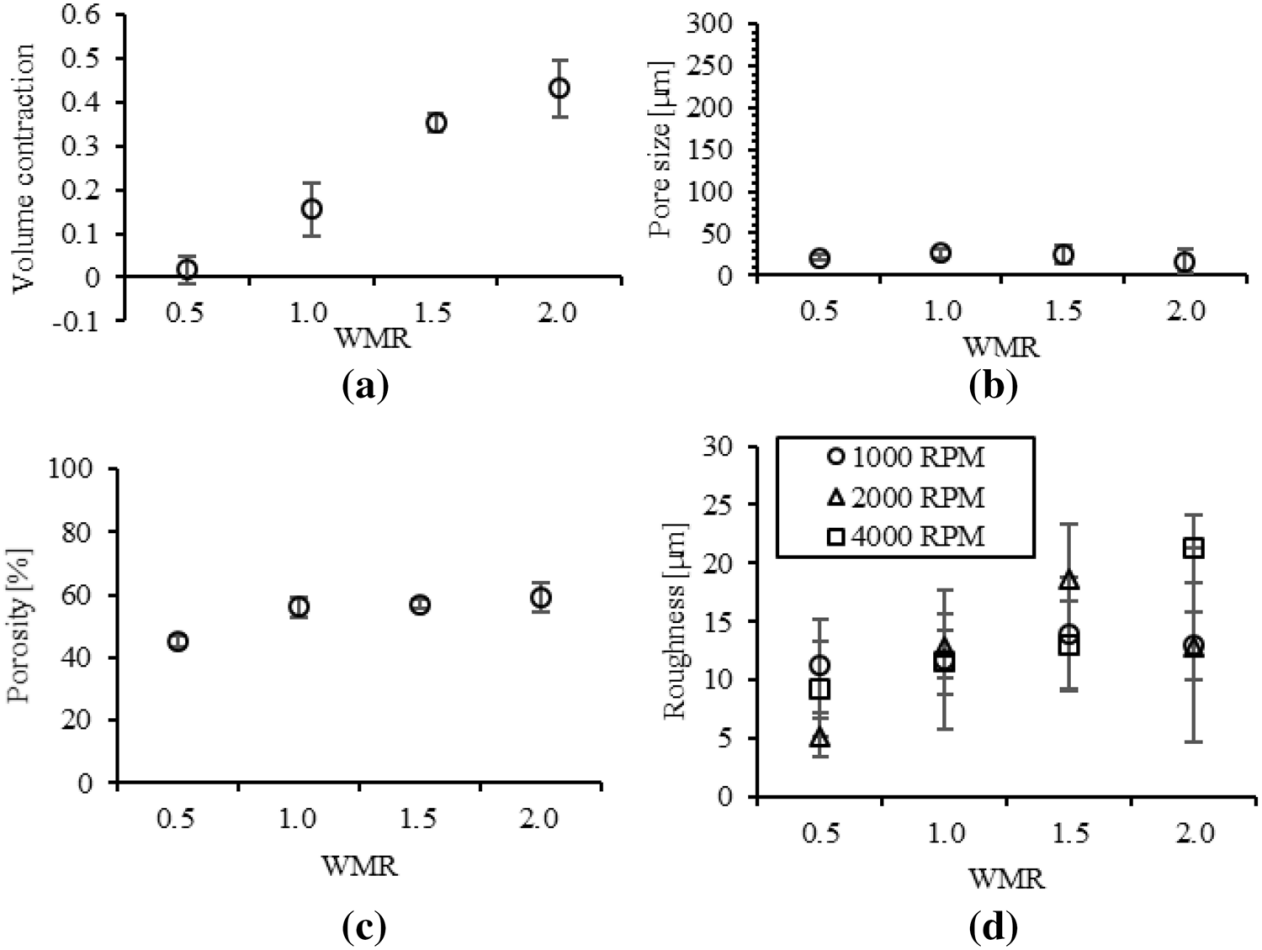
Properties of the present porous PDMS layer, fabricated at t_e_ = 150 °C for varying WMR: (**a**) volumetric contraction; (**b**) pore size; (**c**) porosity; (**d**) surface roughness.

The present porous PDMS layer is volumetrically contracted during the fabrication process of the citric acid crystal removal. The volumetric contraction of the present porous PDMS layer is measured for varying t_e_ and WMR to verify the effect of the volumetric contraction on the pore size, pore size uniformity, and porosity. The volumetric contractions are dramatically decreased from 0.21 ± 0.037 µm to 0.04 ± 0.035 µm, fabricated at t_e_ in the range of 60–70 °C and 120 ~ 150 °C, respectively (Fig. 2a). The volumetric contractions are increased depending on WMR (Fig. 3a). The volumetric contractions, fabricated at WMR = 0.5, 1.0, 1.5, and 2.0 are 0.03 ± 0.023, 0.16 ± 0.060, 0.35 ± 0.021, and 0.43 ± 0.064, respectively.

### Pore size and shape

The two solvent of the porous PDMS layer, ethanol (boiling point = 78.4 °C) and toluene (boiling point = 110.6 °C), are both boiled over the temperature of 120 °C. t_e_ over 120 °C increases the evaporation speed of the solvent mixture of the ethanol and the toluene by boiling, thus reducing the pore size and increasing the pore size uniformity. The pore sizes are 160 ± 67 µm and 20 ± 2.9 µm, fabricated at t_e_ in the range of 60–70 °C and 120–150 °C, respectively (Fig. 2b). The pore size uniformity based on coefficient of variance (CV) are 0.41 and 0.14, fabricated at t_e_ in the range of 60–70 °C and 120–150 °C, respectively (Table 1). The pore shape of the present porous PDMS layer, fabricated at t_e_ below 70 °C, which is the temperature under the boiling point of the ethanol and the toluene solvent mixture, is irregular due to the slow evaporation speed. The pore shape of the present porous PDMS layer, fabricated at t_e_ over 120 °C, which is the temperature over the boiling point of the ethanol and the toluene solvent mixture, is spherical due to the fast evaporation speed (Fig. s2).

**Table 1:** Pore size of the present porous PDMS layer fabricated at WMR = 0.5 for varying t_e_.

t_e_** [°C]	Pore size [µm]	Pore size CV*
60	200 ± 52	0.27
70	130 ± 67	0.50
120	19 ± 2.2	0.12
130	18 ± 2.4	0.13
140	18 ± 3.1	0.17
150	21 ± 2.9	0.14

*Coefficient of variance (CV) = standard deviation / average.

**Toluene and ethanol solvent mixture evaporation temperature (t_e_), effective for citric acid crystal dimensions.

**Figure 4:**
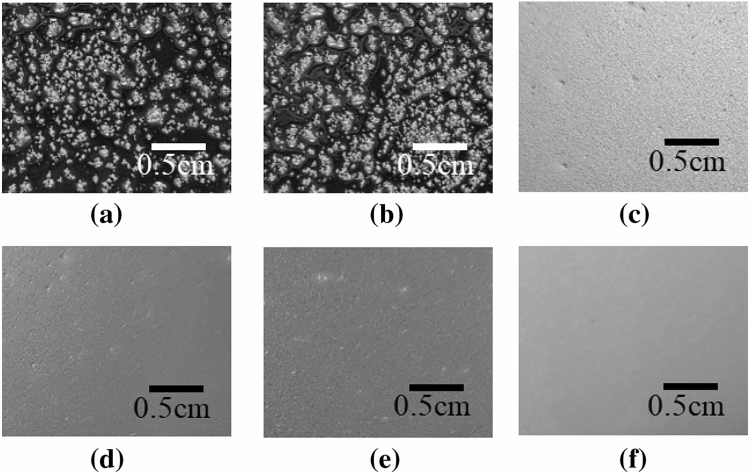
Surface of the spin-coated (2000 rpm) present porous PDMS layer, fabricated at WMR = 0.5 for varying t_e_: (**a**) 60 °C; (**b**) 70 °C; (**c**) 120 °C; (**d**) 130 °C; (**e**) 140 °C; (**f**) 150 °C.

The present porous PDMS layer requires the uniform and small size citric-acid crystals for the formation of the porous PDMS membrane with the uniformly distributed small-sized pores, by spin-coating. The porous PDMS layer, fabricated at t_e_ = 150 °C, shows the smallest pore size and the pore size CV as 19 ± 17 µm and 0.08, respectively, thereby enabling the formation of the uniformly distributed small-sized pores in the spin-coated porous PDMS layer. t_e_ = 150 °C is decided as the optimal fabrication condition for the spin-coating process of the present porous PDMS layer. The surface of the spin- coated porous PDMS layers, fabricated at WMR = 0.5 for varying t_e_ are shown in Fig. 4.

The pore size of the present porous PDMS layer is dominantly affected by not WMR but t_e._ The pore size of the present porous PDMS layer is similar for varying WMR. The pore sizes of the present porous PDMS layers, fabricated at t_e_ = 150 °C for WMR = 0.5, 1.0, 1.5, and 2.0 are 21 ± 2.9 µm, 26 ± 5.6 µm, 20 ± 10 µm, 20 ± 13 µm, respectively (Table 2). The pore size uniformity of the present porous PDMS layer is affected by the pore shape distortion. The porous PDMS layer with high WMR is easy to be collapsed due to the low volume of PDMS, mechanical supports in the present porous PDMS layer, thereby the higher WMR leads higher volumetric contraction in the present porous PDMS layer fabrication. The pore shape (Fig. s3) of the present porous PDMS layer is distorted by increment of WMR due to the volumetric contraction. The pore distortion level is quantified by the division of the pore dimensions of x and y directions (Fig. s4). The pore size CV of the present porous PDMS layers, fabricated at t_e_ = 150 °C for WMR = 0.5, 1.0, 1.5, and 2.0 are 0.13, 0.22, 0.42, and 0.79, respectively (Fig. 3b), thereby the pore size uniformity is decreased depending on WMR. The pore size uniformity is affected by the distortion of the pore shape resulted from the volumetric contraction of the present porous PDMS layer.

**Table 2:** Pore size of the present porous PDMS layer fabricated at t_e_ = 150 °C for varying WMR.

WMR*	Pore size [µm]	Pore size CV
0.5	18 ± 2.4	0.14
1.0	26 ± 5.6	0.21
1.5	20 ± 10	0.42
2.0	20 ± 13	0.79

*PDMS and citric acid weight mixing ratio (WMR = Citric acid mass / PDMS mass), effective for porosity.

### Porosity

The porosity of the present porous PDMS layer is affected by the volumetric contraction. The porosity is the ratio of the volume of the PDMS and the volume of the pores in the present porous PDMS layer. The volumetric contraction reduces the volume of the pores, thereby affecting the porosity of the present porous PDMS layer. The experimental porosity of the present porous PDMS layer, fabricated at WMR = 0.5 for varying t_e_ is shown in Fig. 2c with the theoretical porosity of neglecting and considering the volumetric contraction (Table s1). The experimental porosity of the present porous PDMS layer, fabricated at t_e_ = 150 °C for varying WMR is shown in Fig. 3c with the theoretical porosity of neglecting and considering the volumetric contraction (Table s2). The experimental porosity of the present porous PDMS layer follows the theoretical porosity considering the volumetric contraction. Thus, the porosity of the present porous PDMS layer is affected by the volumetric contraction.

### Water vapor transmission rate

**Figure 5:**
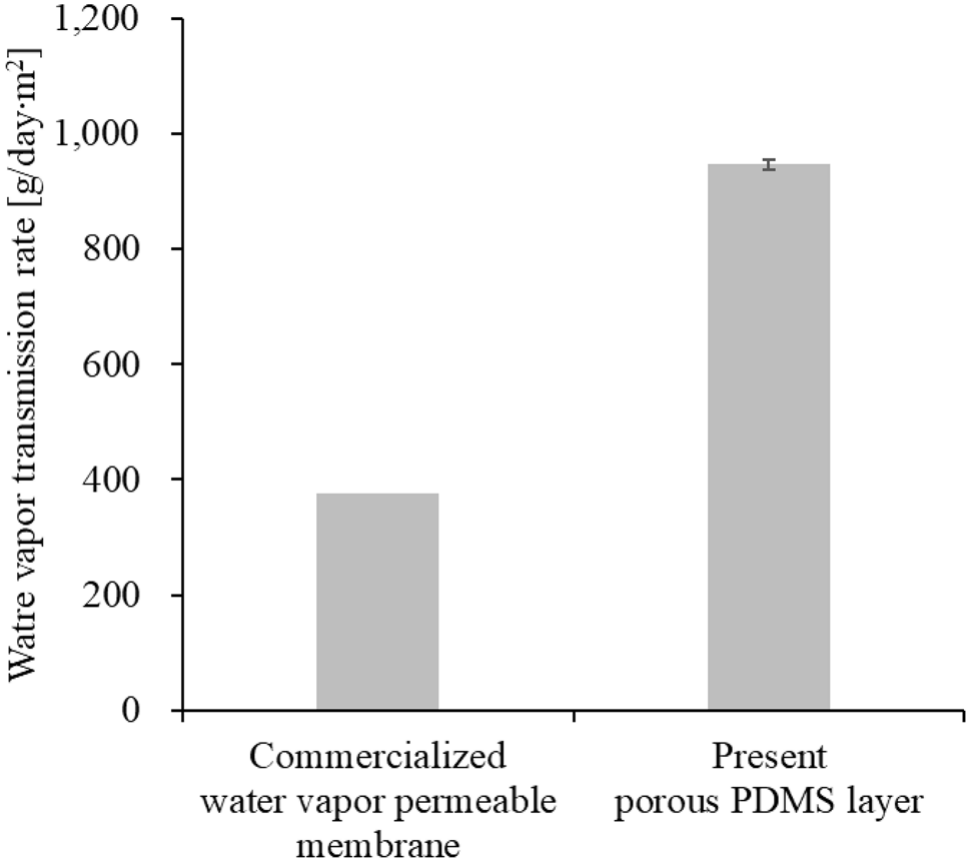
Water vapor transmission rate of the commercialized water vapor permeable membrane (Opsite flexfix, Smith & nephew) and the present porous PDMS layer, fabricated at t_e_ = 150 °C and WMR = 0.5.

The present porous PDMS layer requires the water vapor transmission rate over 432 g/day·m^2^^3^ to reduce the skin trouble. The theoretical water vapor transmission rate of the present porous PDMS layer, fabricated at WMR = 0.5, 1.0, 1.5, and 2.0 is 774, 652, 571, 573 g/day·m^2^, respectively, in the thickness of 27.6 µm, thereby showing the highest water vapor transmission rate at the fabrication condition of WMR = 0.5 (Fig. s5). The specific theoretical water vapor transmission rate calculation method is shown in the methods section. The experimental water vapor transmission rates of the conventional PDMS, the present porous PDMS layers fabricated at WMR = 0.5, and the present porous PDMS layers fabricated at WMR = 2.0 are 296 ± 2.7 g/day·m^2^, 947 ± 8.7 g/day·m^2^ and 751 ± 6.9 g/day·m^2^, respectively at the temperature of 30 °C and relative humidity of 11%RH. The thickness of the conventional PDMS, the present porous PDMS layers fabricated at WMR = 0.5, and the present porous PDMS layers fabricated at WMR = 2.0 are 27.6 ± 0.11 µm, 31.2 ± 0.74 µm, and 26.1 ± 0.71 µm. The present porous PDMS layer fabricated at WMR = 0.5 shows the highest water vapor transmission rate among the fabrication conditions tried in the research. WMR = 0.5 is decided as the optimal fabrication condition for the daily life skin attachable device by reducing the skin trouble. The present porous PDMS layer, fabricated at t_e_ = 150 °C and WMR = 0.5, shows the 2.5 times higher water vapor transmission rate (Fig. 5) compared to the water vapor transmission rate (377 g/day·m^2^ at the thickness of 70 µm) of the commercialized water vapor permeable membrane (Opsite flexfix, Smith & nephew)^16^. The present porous PDMS layer enables the skin trouble reduction compared to the conventional water vapor permeable membranes by the improvement of the water vapor transmission rate. The water vapor transmission rate (947 ± 8.7 g/day·m^2^) of the present porous PDMS layer, fabricated at WMR = 0.5 and t_e_ = 150 °C, with no skin attachment is 2.2 times higher than that of the human skin requirement (432 g/day·m^2^^3^), therefore the attachment of the present porous PDMS layer on human skin do not disturb the water vapor evaporation of the human skin.

### Optimal fabrication condition

WMR = 0.5 and t_e_ = 150 °C is decided as the optimal fabrication condition considering the water vapor permeability and the spin-coating possibility for the uniformly distributed small-sized pores.

### Pore size comparison of the present porous PDMS layer and Non-crystalized porous PDMS layer

**Figure 6:**
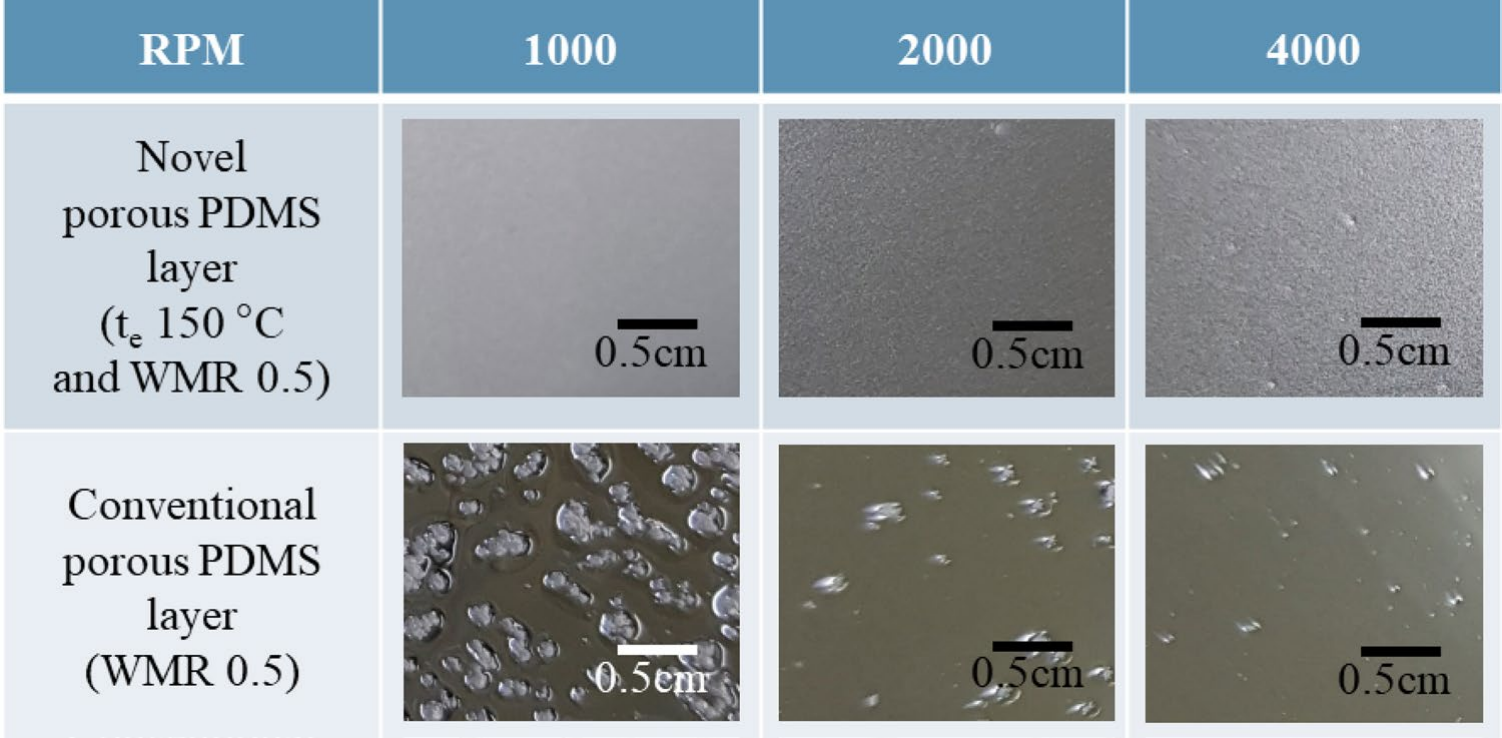
Surface of the present porous PDMS layer and the non-crystallized PDMS layer, respectively fabricated at the spin-coating RPM of 1000, 2000, and 4000.

**Figure 7:**
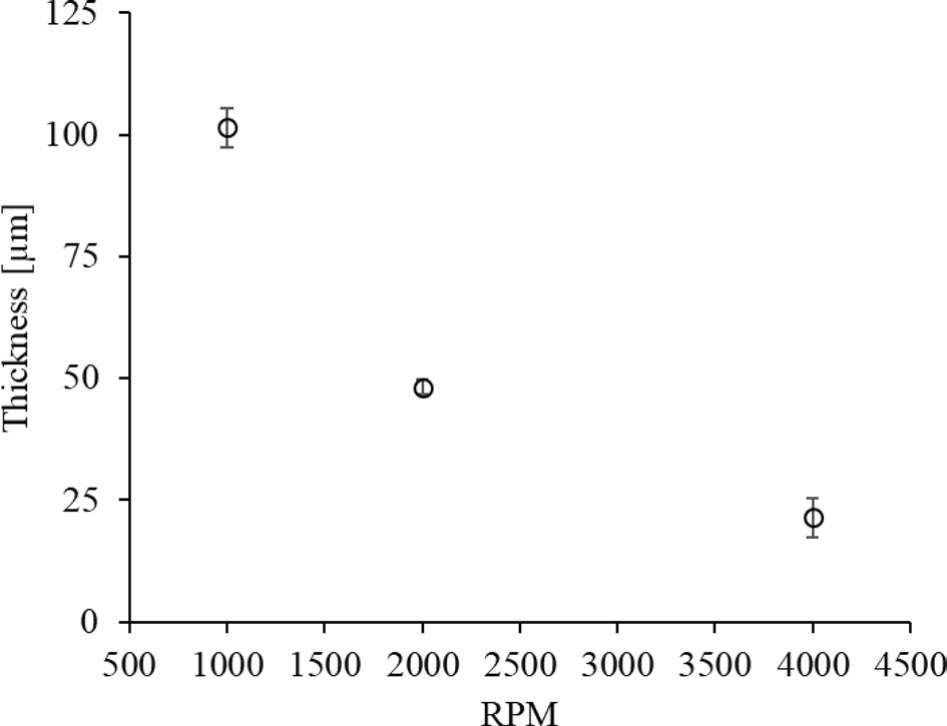
Thickness of the present porous PDMS layer, fabricated at t_e_ = 150 °C and WMR = 0.5, depending on the spin-coating RPM.

The conformal skin contact of the porous PDMS layer is important for the accurate daily-life physiological signal monitoring. The conformal skin contact of the porous PDMS layer is enabled by the fabrication of the spin-coated porous PDMS layer with uniform and small size citric acid crystals. The uniform and small size citric acid crystals prevents the removal of the citric acid particles due to the centrifugal force during the spin-coating process, thereby forming the uniform and small size pores in the porous PDMS layer. The pore size and the pore size CV of the present porous PDMS layer fabricated at t_e_ = 150 °C and WMR = 0.5 are compared to those of the non-crystallized porous PDMS layer, fabricated at WMR = 0.5 to verify the present porous PDMS layer’s applicability for the conformal skin attachable material. The non-crystallized porous PDMS layer is fabricated by the citric acid mixing and dissolution without the crystallization process. The pore size (19 ± 1.7 µm) of the present porous PDMS layer is 93.2%p decreased compared to that (280 ± 117 µm) of the non-crystallized porous PDMS layer. The pore size uniformity (pore size CV = 0.008) of the present porous PDMS layer is 425%p increased compared to that (pore size CV = 0.42) of the non-crystallized porous PDMS layer (Table 3). The surface of the spin-coated present porous PDMS layer and non-crystallized PDMS layer, fabricated at the spin-coating RPM of 1000, 2000, and 4000 are shown in Fig. 6. The present porous PDMS layer is applicable to the skin attachable device by distributing the uniform and small size pores in the porous PDMS layer in the thickness range of 21–101 µm (Fig. 7).

**Table 3:** Pore size and CV of the present and the non-crystallized porous PDMS layer.

	Pore size [µm]	Pore size CV
Present porous PDMS layer (t_e_ = 150 °C and WMR = 0.5)	21 ± 2.9	0.14
Non-crystallized PDMS layer* (WMR = 0.5)	280 ± 117	0.42

*Non-crystallized porous PDMS layer is fabricated by mixing PDMS and citric acid without crystallization.

### Water vapor transmission rate comparison of the present porous PDMS layer and PDMS layer with holes array

The water vapor transmission rate of the present porous PDMS is compared to the conventional PDMS layer with holes array. The holes array design is shown in Fig. s8a,b. The thickness of the present porous PDMS layer and the conventional PDMS layer with holes array is designed as 31.2 µm. The conventional PDMS layer with holes array has the same water vapor transmission rate with the present porous PDMS layer at the hole radius of 83.4 µm. The holes occupy the 54.6% of the overall area, thereby reducing the device integration efficiencies. Young’s modulus of the conventional PDMS layer with 83.4 µm radius holes array is 307.5 kPa which is 65% of that of the present porous PDMS layer (Fig. s9), thereby having lower durability than the present porous PDMS layer.

### Surface roughness

The surface roughness of the present porous PDMS layer is characterized for the reliability verification of the human physiological sensor integration on the present porous PDMS layer. The surface roughness of the present porous PDMS layer fabricated at WMR = 0.5 for varying t_e_ is shown in Fig. 2d. The surface roughness of the present porous PDMS layer fabricated at t_e_ = 150 °C for varying WMR is shown in Fig. 3d. The surface roughness of the present porous PDMS layer is lowest as 5.1 ± 0.19 µm in the fabrication conditions of t_e_ = 150 °C and WMR = 0.5. Thereby the fabrication condition of t_e_ = 150 °C and WMR = 0.5 has the highest reliability for the human physiological sensor integration on the present porous PDMS layer.

### Endurance on the human skin use

The endurance of the present porous PDMS layer is expected by applying strains in the human skin strain range of 0.3^17^. The stress–strain curve of the porous PDMS layers (Fig. s5) are linear in the human skin strain range, thereby the present porous PDMS layers are elastic material with the human skin attachment applications. Furthermore, the water vapor transmission rate of the porous PDMS layer do not show the change with the strain in the human skin range (Fig. s5). The water vapor transmission rate of the present porous PDMS layer, fabricated at WMR = 0.5 and t_e_ = 150 °C, are 0.0295 g m/day m^2^, 0.0290 g m/day m^2^, and 0.0293 g m/day m^2^ at the strain of 0%, 0.2%, and 0.4%, respectively. Therefore, the present porous PDMS layer is able to be adapted on the repeated use on the human skin attachment applications.

### Contact angle

The contact angle (110°) of the present porous PDMS layer fabricated in the conditions of WMR = 0.5 and t_e_ = 150 °C is similar with that (108°) of the non-porous PDMS layer (Fig. s6). The similar contact angles of the present porous PDMS and the non-porous PDMS indicate that the present porous PDMS layer fabrication method causes minor chemical changes on the surface of the PDMS. Therefore, the water vapor transmission rate improvement of the present porous PDMS layer comes from structural effect dominantly.

### Thermal conductivity

The thermal conductivities of the present porous PDMS layer, fabricated at WMR = 0.5 and t_e_ = 150 °C and the conventional non-porous PDMS layer are 0.219 ± 0.0036 W/(m K) and 0.166 ± 0.0012 W/(m K), respectively. The present porous PDMS layer shows the 32%p higher thermal conductivities than the conventional non-porous PDMS layer, thereby having better thermal dissipation ability than the conventional non-porous PDMS layer.

### Human skin attachment test

The skin trouble reduction ability of the present porous PDMS layer is verified by the human skin test. The present porous PDMS layer and the conventional non-porous PDMS layer are attached on the same human skin subject (n = 1) for 7 days. The skin attaching the conventional non-porous PDMS layer changes into red color. However, the skin attaching the present porous PDMS layer does not show the severe color change (Table 4). The dermatological diagnosis of skin redness by the attachment of the conventional non-porous PDMS layer is ‘contact dermatitis’ which is coming from the lack of water vapor evaporation on human skin. Thereby, the improvement of the water vapor transmission rate of the present porous PDMS layer reduces the skin troubles of the human skin attachment.

**Table 4:** Human skin attachment test of the conventional and present porous PDMS layer.

	Conventional non-porous PDMS layer	Present porous PDMS layer (t_e_ = 150 °C and WMR = 0.5)
Skin redness		
Redness level* (Redness level of surround skin)	8 ± 2.8 (21 ± 4.0)	20 ± 3 (22 ± 4.1)
Skin redness index**	2.6	1.1
Itchiness level***	3	0
Medical diagnosis (reason)	Irritant contact dermatitis (low water vapor permeability)	N/A

*Redness level is measured on 6 spots of each specimen with HSL color code (0: red, 25: yellow).

**Skin redness index = Redness level of the specimen attached skin / Redness level of surround skin.

***Itchiness level survey (0: normal state, 6: mosquito biting.

All results are measured followed by 7 days of the specimens attachment on 1 subject.

In summary, the present research experimentally verifies the novel porous PDMS layer fabricated by controlling the citric acid crystallization conditions of t_e_ and WMR for: i) enabling the spin-coated porous PDMS layer with the uniformly distributed small-sized pores for the conformal skin contact; ii) improving the water vapor transmission rate for the skin trouble reduction at the daily life skin contact.

The present porous PDMS layer shows the smallest pore size and the pore size CV at the fabrication condition of t_e_ = 150 °C. The present porous PDMS layer shows the highest water vapor transmission rate at the fabrication condition of WMR = 0.5. The present porous PDMS layer fabricated at t_e_ = 150 °C and WMR = 0.5 is effective for the conformal contact and the skin trouble reduction of the skin attachable devices. The present porous PDMS layer fabricated at t_e_ = 150 °C and WMR = 0.5 decreases the pore size 93.2%p, and increases the pore size uniformity 425%p compared to the conventional porous PDMS layer fabrication method. The present porous PDMS layer, fabricated at t_e_ = 150 °C and WMR = 0.5, forms the membrane in the thickness of 21 ~ 101 µm by spin-coating for the skin attachable application. The present porous PDMS layer, fabricated at t_e_ = 150 °C and WMR = 0.5, has the 2.2 times higher water vapor transmission rate (947 ± 10.8 g/day m^2^) compared to the water vapor evaporation rate of the human skin, thereby enabling the effective water vapor evaporation on human skin for the skin trouble reduction. The present porous PDMS layer is applicable to the human daily-life skin attachable devices.

## Materials and methods

### The amount of the material amount used in the present porous PDMS layer fabrication

The amounts of the citric acid and the PDMS are decided by WMR. The volume of the ethanol is decided as the volume saturated by the citric acid. The volume of the toluene volume is decided as same as the volume of the ethanol.

### *t*_*e*_* range*

t_e_ range of the present porous PDMS layer is limited as below 70 °C or between 120–153 °C. t_e_ in the range of 80 and 110 °C crystalize the citric acid not in micrometer-dimensions but in centimeter-dimensions due to the vapor pressure differences between the ethanol^18^ and the toluene^19^ (Fig. s8). t_e_ over 153 °C does not form pores by melting the citric acid crystals^20^. Therefore, the present research controls t_e_ on 60, 70, 120,130, 140, and 150 °C for the present porous PDMS layer fabrication (Fig. s9).

### Volumetric contraction measurement method

The volumetric contraction is measured 3 times for the 5 specimens of the present porous PDMS layer for the fabrication conditions of varying t_e_ and WMR. The volumetric contraction is calculated using the equation of

$$ {\text{C}} = \frac{{V_{b} - V_{a} }}{{V_{b} }} $$

where C is volumetric contraction, $V_{b}$ is specimen volume before the citric acid removal, and $V_{a}$ is specimen volume after the citric acid removal.

### Pore size and shape measurement method

The pore size is measured with 20 pores for 2 specimens of the present porous PDMS layer for the fabrication conditions of varying t_e_ and WMR, using scanning electron microscope (SEM).

### Porosity measurement method

The porosity is measured with the 5 specimens of the present porous PDMS layer for the fabrication conditions of varying t_e_ and WMR. The porosity is calculated using the equation of

$$ p = \frac{{V_{p} }}{{V_{p} + V_{m} }} $$

where $p$ is porosity, ${\varvec{V}}_{{\varvec{p}}}$ is volume of the pores, and ${\varvec{V}}_{{\varvec{m}}}$ is the volume of the material. The volume of the pores is estimated by the mass difference of the same volume of the present porous PDMS layers and the conventional PDMS layers without pore.

### Surface roughness measurement method

The surface roughness are measured 3 times for 2 specimens of the present porous PDMS layer for the fabrication conditions of varying t_e_ and WMR. The spin coating thickness and the surface roughness is measured by the surface stylus profiler (p-6, KLA Tencor, US). The stylus is moved 2000 µm with the velocity of 10 µm/sec for profiling the surface of the present porous PDMS layer. The surface roughness is calculated with the equation^21^ of

$$ \begin{aligned} p & = \mathop {\max }\limits_{i} y_{i} \\ v & = \mathop {\min }\limits_{{\varvec{i}}} {\varvec{y}}_{{\varvec{i}}} \\ {\varvec{R}}_{{{\varvec{p}} - {\varvec{v}}}} & = {\varvec{p}} - {\varvec{v}} \\ \end{aligned} $$

where ${\varvec{y}}_{{\varvec{i}}}$ is deviation of the assessed profile, ${\varvec{p}}$ is maximum peak height, ${\varvec{v}}$ is maximum valley depth, and ${\varvec{R}}_{{{\varvec{p}} - {\varvec{v}}}}$ is roughness.

### Young’s modulus measurement method

Young’s modulus is measured with 4 specimens for the present porous PDMS layer fabricated at t_e_ = 150 °C and WMR = 0.5. Young’s modulus measurement specimens are fabricated by laser cutting of the present porous PDMS layers. The stress–strain curves are plotted with a tensile testing machine (instron 8818, instron, UK) in the condition described in Table s3. Figure s12 shows the specimen designs for Young’s modulus test. Figure s13 shows the experimental setup for Young’s modulus test.

### Water vapor transmission rate measurement method

The water vapor transmission rate is measured 4 times. The water vapor permeability is measured by wet cup method of ASTM E 95–96 (Fig. s14) in the constant temperature and humidity chamber. The chamber temperature and humidity are controlled as 30 °C and 11%RH, respectively. The water vapor transmission rate is calculated with the equation^22^ of

$$ {\text{Water}}\,{\text{vapor}}\,{\text{transmission }}\,{\text{rate}} = \frac{{{\text{Mass}}\,{\text{H}}_{2} {\text{O}}\,{\text{lost}}}}{{{\text{Time}} \times {\text{area}}}} $$

The theoretical water vapor transmission rate is calculated considering the water vapor transmission rate of the conventional PDMS, the law of mixture^23^ and the pore distortion level.

### Contact angle

Contact angle is measured by two specimens for the present porous PDMS layer fabricated at t_e_ = 150 °C and WMR = 0.5. The contact angles are measured with a contact angle analyzer (Phoenix, SEO, Republic of Korea).

### Thermal conductivity

The thermal conductivity is measured 3 times. The thermal conductivity is calculated with the equations of

$$ {\text{Thermal conductivity}} = {\text{Density }} \times {\text{ Thermal diffusivity }} \times {\text{ specific heat}} $$

The thermal diffusivity is measured by LFA447 (Netzsch, German). The specific heat is measured by DSC204F1 Phoenix (Netzsch, German).

### Human skin test

The human skin attachment test is performed with a single subject who had no disease, including skin trouble, and all experimental protocols were approved by the KAIST Institutional Review Board (approval ID: KH2011-18) guidelines. Relevant guidelines are used in the study (Declaration of Helsinki). Before the experiments, all subjects receive detailed explanation of the human experiments and sign the informed consents. The present porous PDMS layer fabricated with the optimal condition and the conventional PDMS layer are attached on the subject dorsal lower arm for 7 days. Skin redness level and itchiness level are measured and surveyed respectively. The skin redness level is measured by photo taken of both PDMS layer attachment spots after the detachment in the conditions of 460 lx illuminance, F/5.6 F-stop, 1/250 s exposure time, and 3200 ISO. The skin redness is measured based on hue index of the hue saturation light (HSL) color code. The hue index 0 and 25 means the red and the yellow, respectively. The skin redness is measured 6 times on the skin of the specimen attached and the skin near the specimen attached, respectively. The skin redness index is calculated by dividing the skin redness of the specimen attached by the skin redness of the skin near the specimen attached. The itchiness level of the subject is surveyed in the range of 0 to 6. 0 means the normal state and 6 means the itchiness level same with the mosquito biting. The reason of the skin redness and the itchiness are analyzed by diagnose from a dermatologist.

## Supplementary information

Supplementary Information.
