# Supplementary material for: Wearable porous PDMS layer of high moisture permeability for skin trouble reduction
Source: Sci Rep. 2021 Jan 13;11:938. doi: 10.1038/s41598-020-78580-z (PMC7806932; doi:10.1038/s41598-020-78580-z)
Supplement: Supplementary file 1 — Supplementary Information. [file 41598_2020_78580_MOESM1_ESM.docx]

**Wearable Porous PDMS Layer of High Moisture Permeability for Skin Trouble Reduction**

Sunghyun Yoon, Minho Seok, Mookyum Kim, and Young-Ho Cho^*^

Department of Bio and Brain Engineering,

Korea Advanced Institute of Science and Technology (KAIST)

291 Daehak-ro, Yuseong-gu, Daejeon 34141, Republic of Korea

Tel.: +82-42-350-8691 / Fax: +82-42-350-8690 / E-mail: [nanosys@kaist.ac.kr](mailto:nanosys@kaist.ac.kr)

Table s1. Porosity of the present porous PDMS layer, fabricated at WMR = 0.5 for varying t_e_

| t_e_ [ºC] | Experimental porosity^*^ [%] | Theoretical porosity | |
| --- | --- | --- | --- |
|  |  | considering volumetric contraction [%] | neglecting volumetric contraction [%] |
| 60 | 33 ± 3.1 | 41 | 46 |
| 70 | 31 ± 3.5 | 40 |  |
| 120 | 44 ± 2.0 | 45 |  |
| 130 | 42 ± 3.0 | 44 |  |
| 140 | 42 ± 2.9 | 44 |  |
| 150 | 45 ± 1.8 | 46 |  |

* $Porosity= {V_{\mathrm{pore}}}/{(V_{\mathrm{PDMS}}+V_{\mathrm{pore}})}$

Table s2. Porosity of the present porous PDMS layer, fabricated at t_e_ = 150 ºC for varying WMR

| WMR | Experimental porosity [%] | Theoretical porosity | |
| --- | --- | --- | --- |
|  |  | considering volumetric contraction [%] | neglecting volumetric contraction [%] |
| 0.5 | 45 ±1.8 | 46 | 46 |
| 1.0 | 56 ± 3.2 | 60 | 63 |
| 1.5 | 55 ± 2.6 | 62 | 72 |
| 2.0 | 59 ± 4.6 | 66 | 77 |

Table s3. Experimental conditions for Young’s modulus test

| Conditions | Values |
| --- | --- |
| Load cell range | 0 ~ 50N |
| Load cell resolution | 1 μN |
| Actuating speed | 10mm/min |


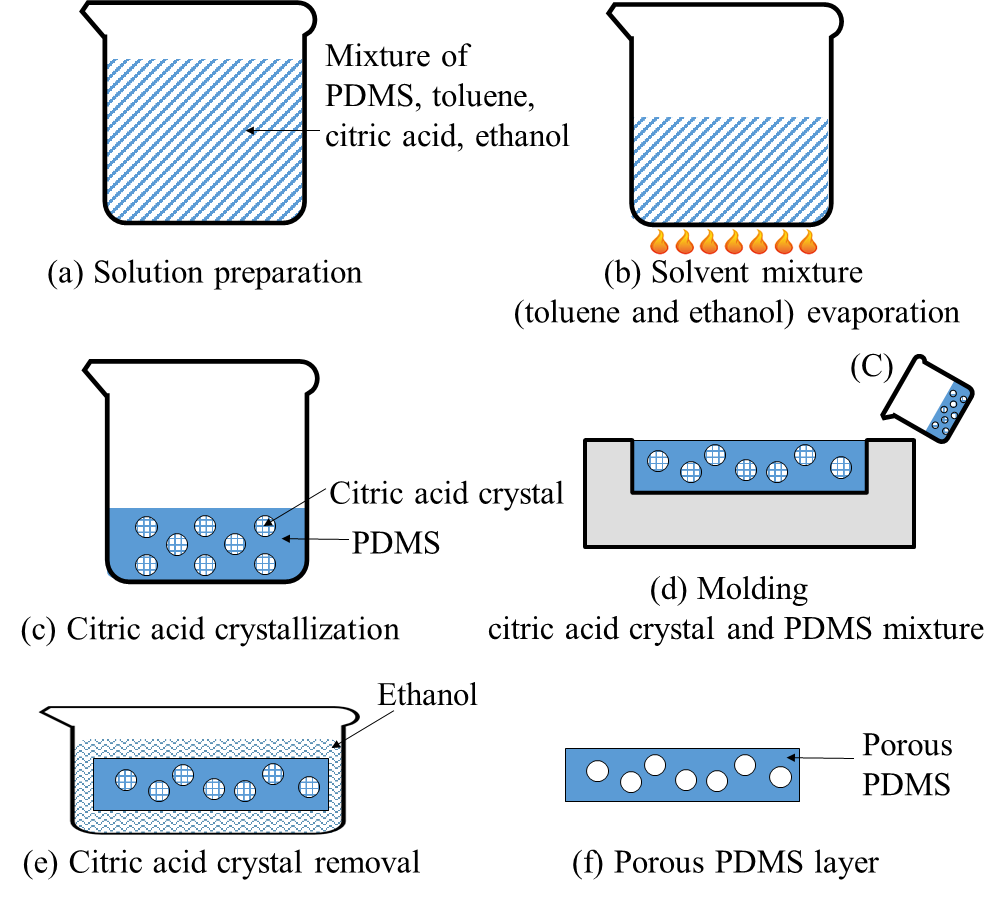


Figure s1. Fabrication process of the present porous PDMS layer


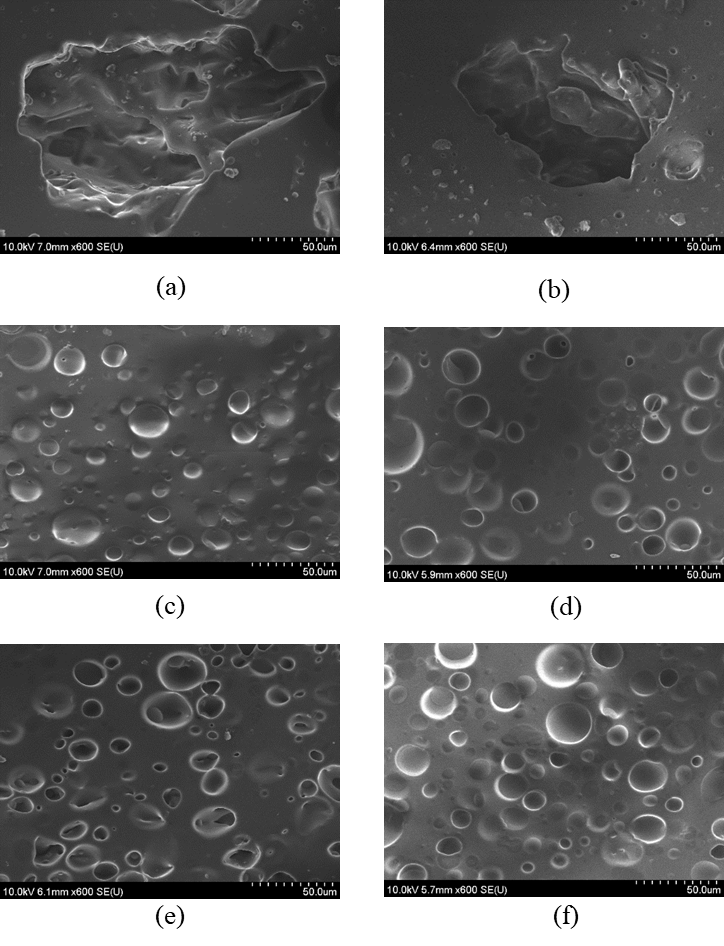


Figure s2. Pore shapes of the present porous PDMS layer, fabricated at WMR = 0.5 for varying t_e_: (a) 60°C; (b) 70°C; (c) 120°C; (d) 130°C; (e) 140°C; (f) 150°C.


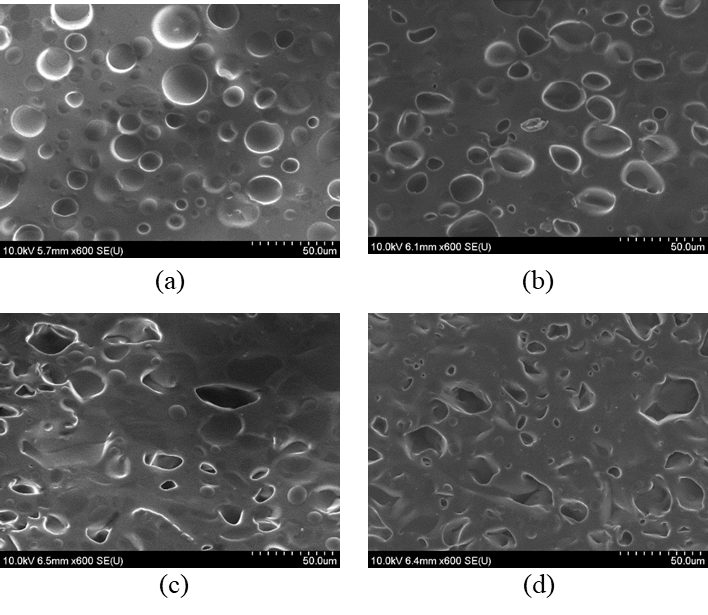


Figure s3. Pores shape of the present porous PDMS layer, fabricated at t_e_ = 150 ºC for varying WMR:
(a) 0.5; (b) 1.0; (c) 1.5; (d) 2.0.


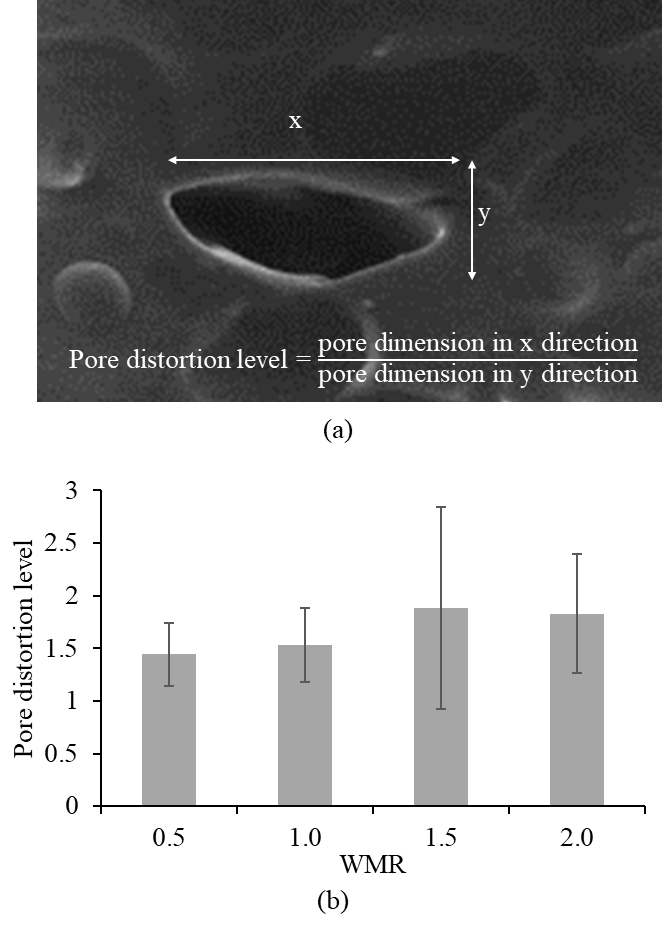


Figure s4. Pore distortion level of the present porous PDMS layer: (a) the pore distortion level, defined from a single pore of the present porous PDMS layer, fabricated at t_e_ = 150 ºC and WMR = 1.5;
(b) pore distortion level for varying WMR


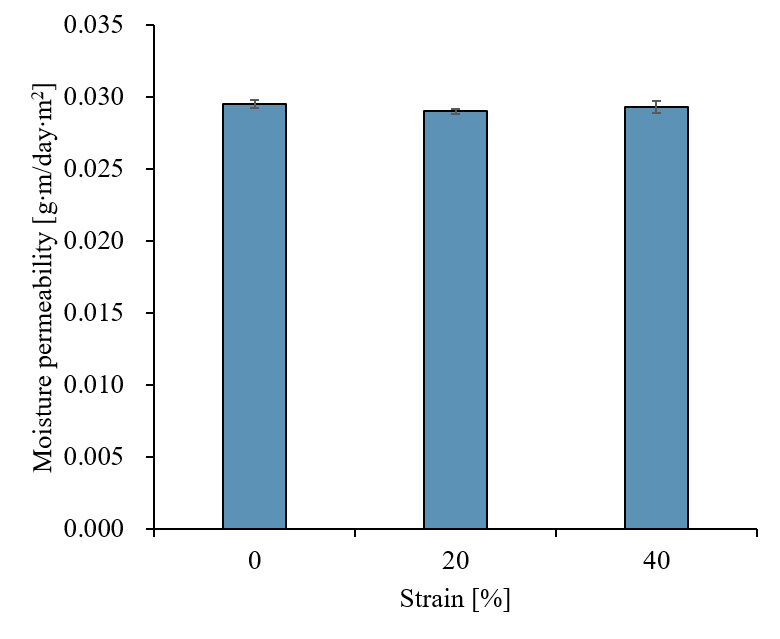


Figure s5. Moisture permeability of the porous PDMS layer on strain


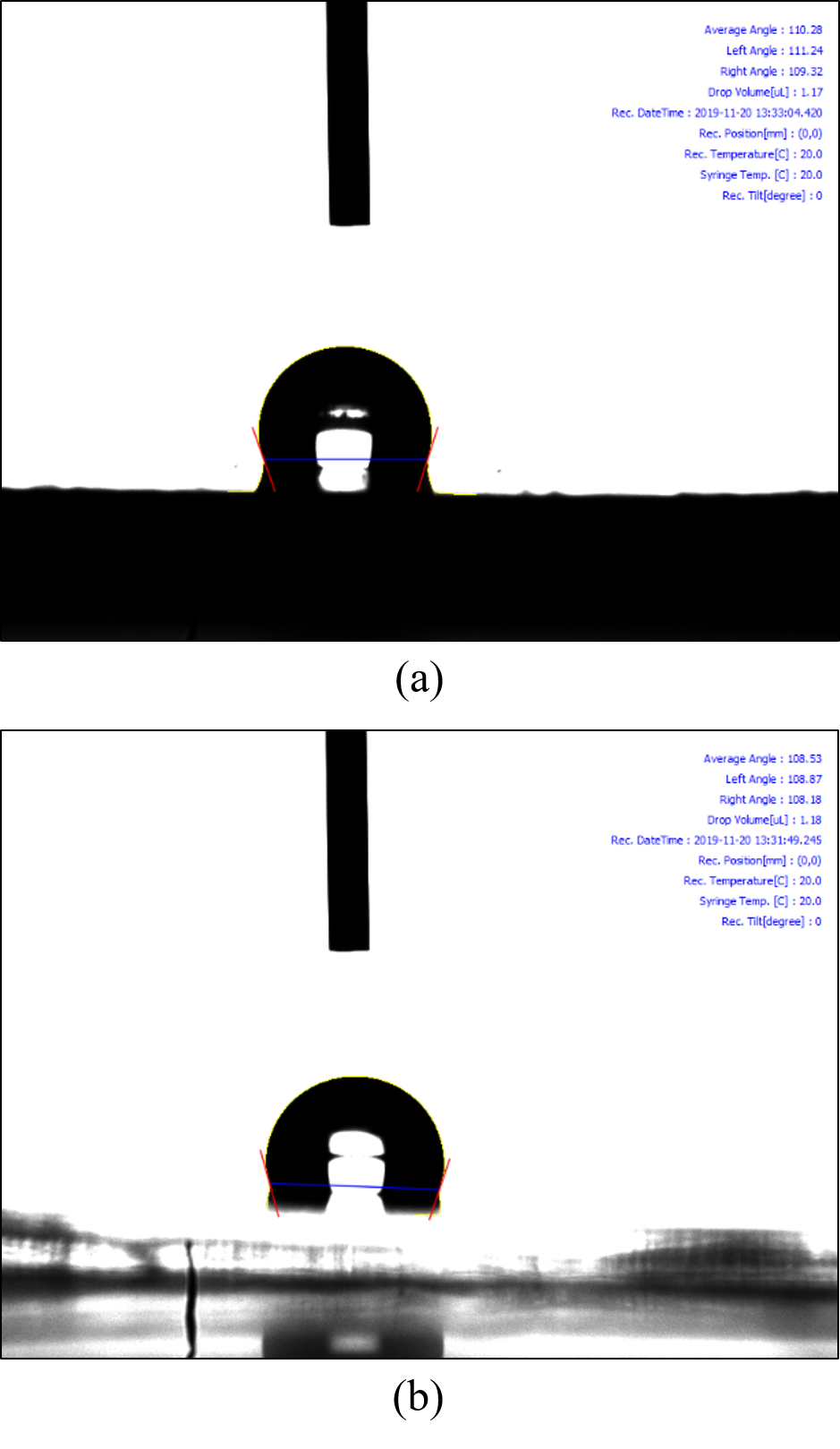


Figure s6. Water contact angles of the corresponding samples: (a) Porous PDMS; (b) Conventional PDMS


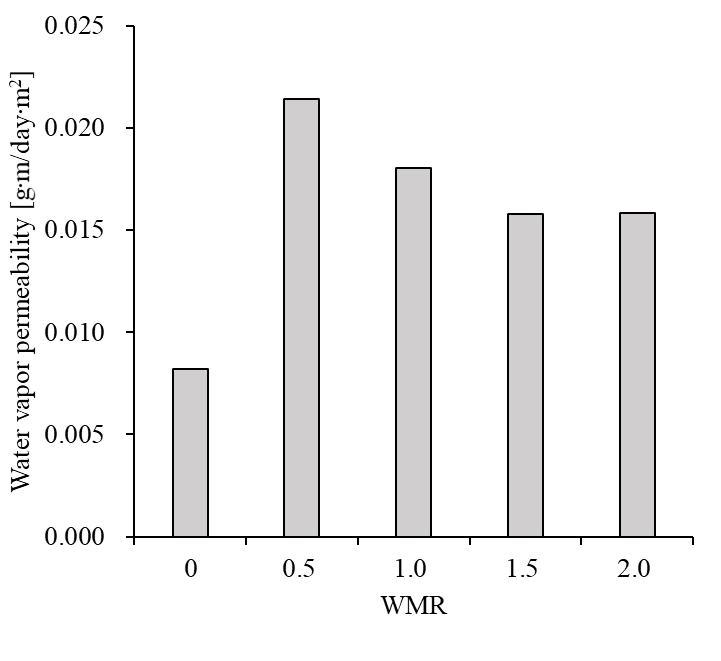


Figure s7. Theoretical water vapor permeability of the present porous PDMS layer, fabricated
at t_e_ = 150 ºC for varying WMR


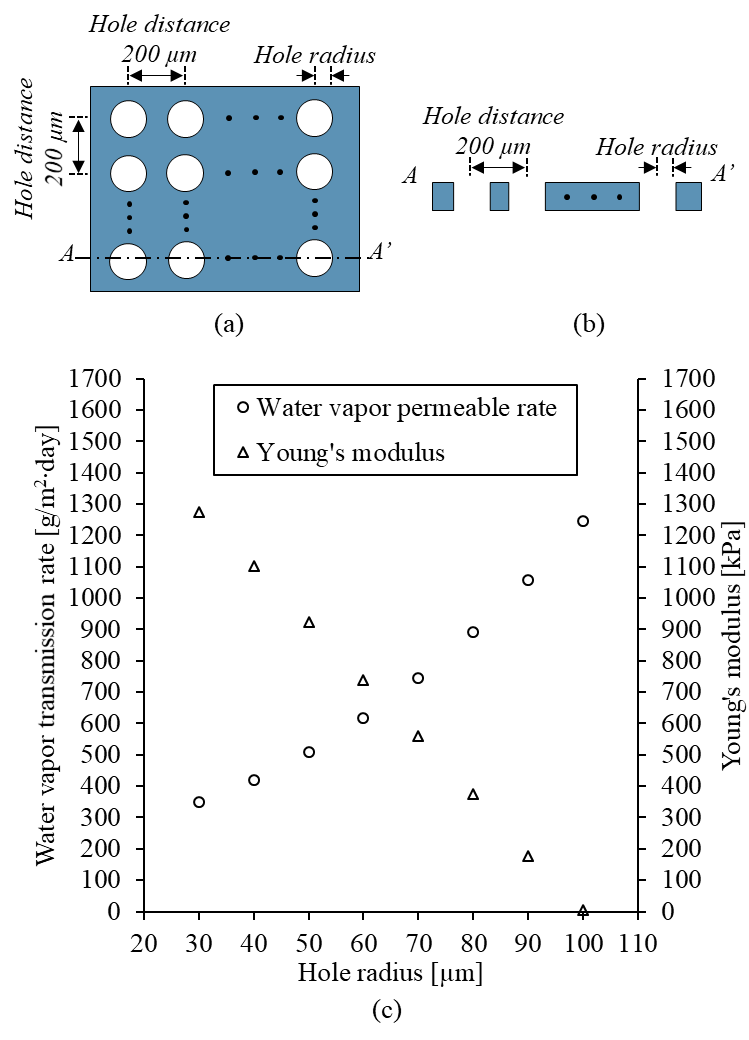


Figure s8. PDMS layer with hole array: (a) top view; (b) cross-sectional view across A-A’ of figure (a);
(c) theoretical water vapor transmission rate and Young’s modulus.


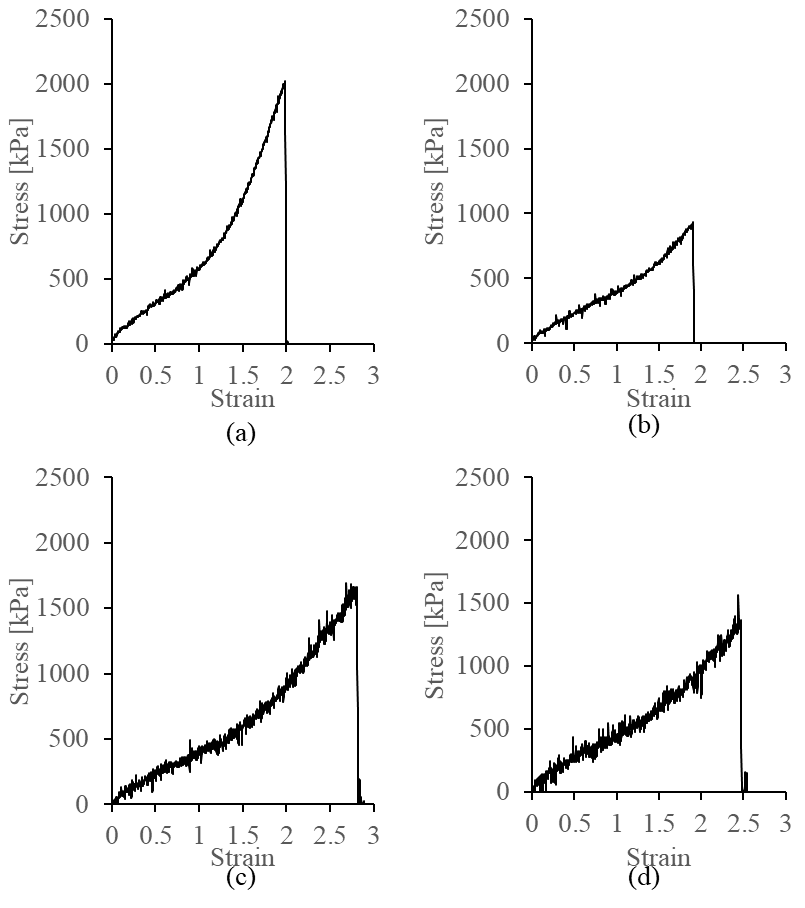


Figure s9. Stress-strain curve of the present porous PDMS layer, fabricated at t_e_ = 150 ºC and:
(a) WMR = 0.5; (a) WMR = 1.0; (a) WMR = 1.5; (a) WMR = 2.0;


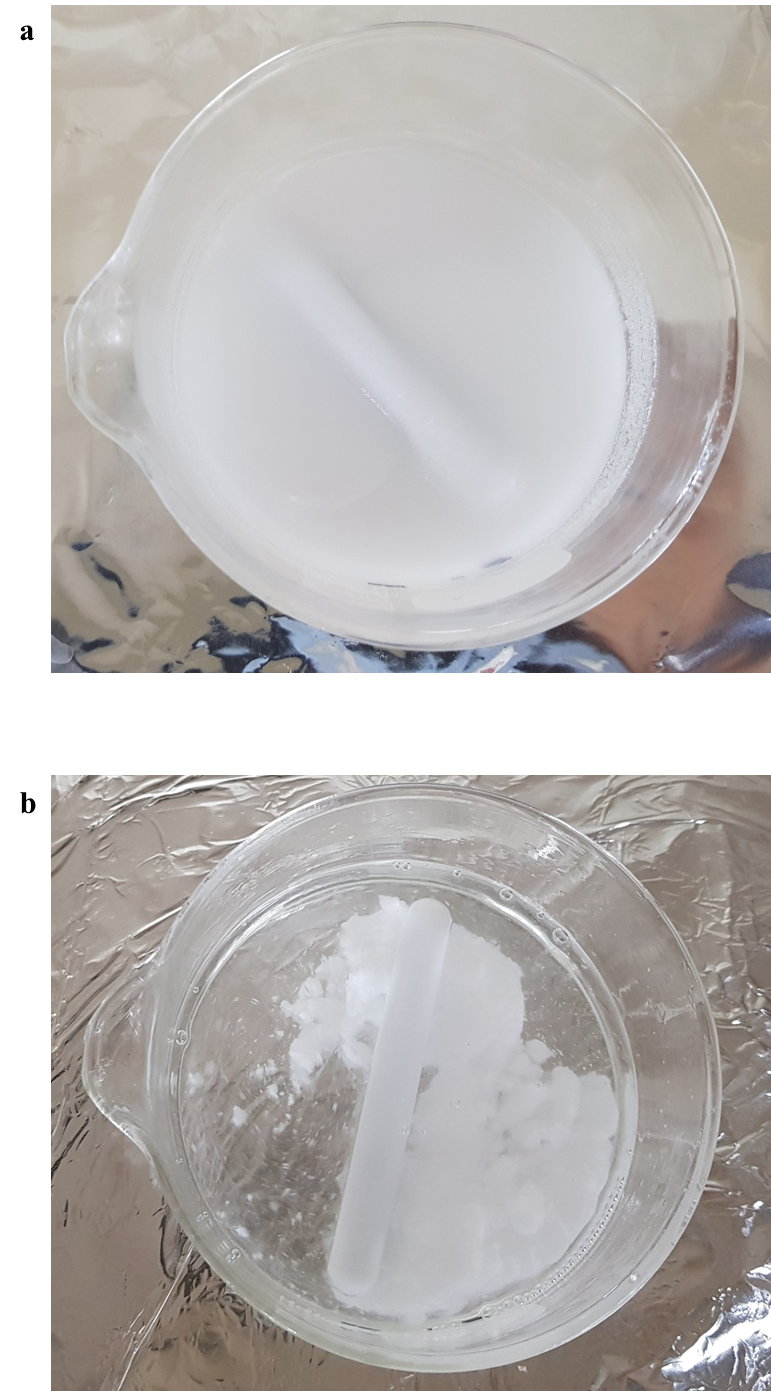


Figure s10. Citric acid crystallization of the mixture of PDMS, toluene, citric acid, and ethanol, fabricated at: a) t_e_ = 150 ºC; b) t_e_ = 90 ºC.


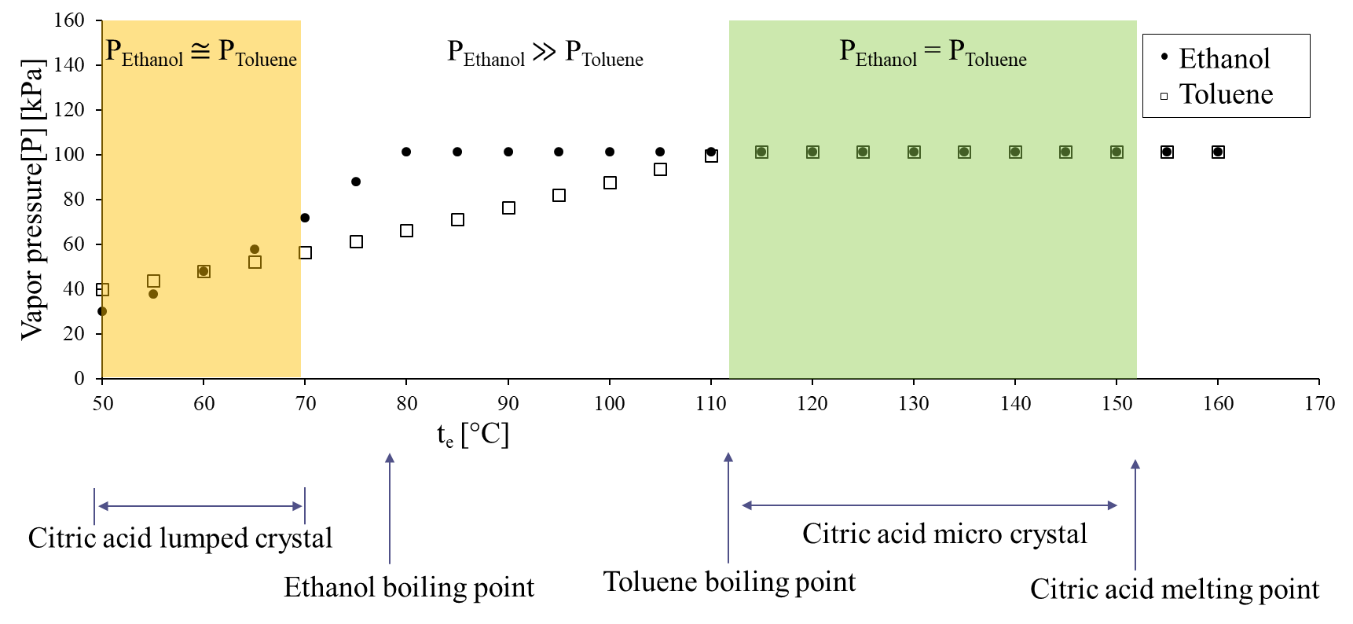


Figure s11. The vapor pressures of the ethanol and the toluene for varying t_e_


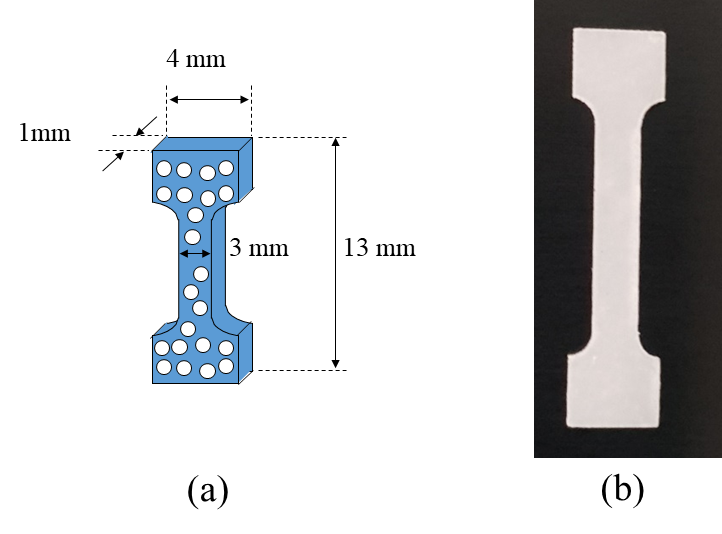


Figure s12. Young’s modulus test specimen: (a) designed; (b) fabricated from the present porous PDMS layer


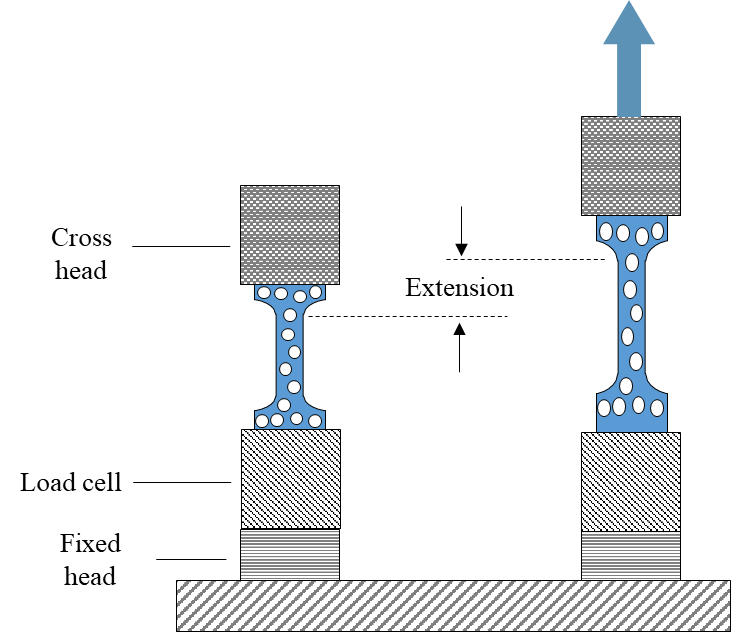


Figure s13. Experimental setup of Young’s modulus test


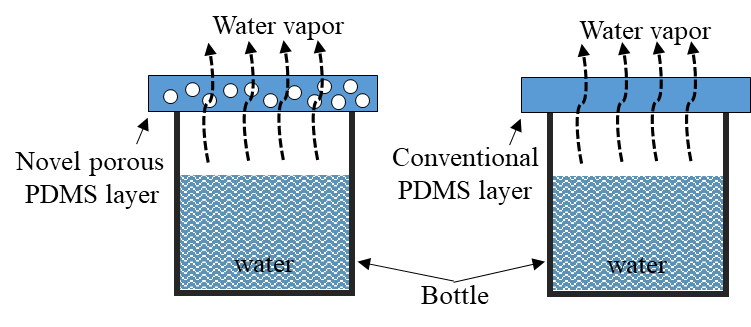


Figure s14. Experimental setup of the water vapor transmission rate measurement for the present porous PDMS layer and the conventional PDMS layer in the constant temperature and humidity chamber
